# Проблема йододефицитных заболеваний В Чеченской республике: оценка текущего состояния и пути решения

**DOI:** 10.14341/probl13306

**Published:** 2023-08-30

**Authors:** Е. А. Трошина, Н. П. Маколина, Н. М. Платонова, М. П. Исаева, Ф. М. Абдулхабирова, Л. В. Никанкина, З. Т. Зураева, У. С. Исаева, Х. В. Атабаева

**Affiliations:** Национальный медицинский исследовательский центр эндокринологии; Национальный медицинский исследовательский центр эндокринологии; Национальный медицинский исследовательский центр эндокринологии; Национальный медицинский исследовательский центр эндокринологии; Национальный медицинский исследовательский центр эндокринологии; Национальный медицинский исследовательский центр эндокринологии; Национальный медицинский исследовательский центр эндокринологии; Республиканский эндокринологический диспансер; Чеченский государственный университет им. А.А. Кадырова; Республиканский эндокринологический диспансер; Чеченский государственный университет им. А.А. Кадырова

**Keywords:** йодный дефицит, заболевания щитовидной железы, йододефицитные заболевания, зоб, йодированная соль, региональная программа профилактики

## Abstract

Широкая распространенность и высокая заболеваемость, скрытое течение, тяжелые инвалидизирующие соматические осложнения, включая когнитивные расстройства, репродуктивные потери и онкопатология, — все это характерно для йододефицитных заболеваний (ЙДЗ) и является серьезным вызовом для системы здравоохранения Российской Федерации, поскольку затрагивает более 3 млн населения страны.ОБОСНОВАНИЕ. Отсутствие на региональном уровне актуальных данных о выраженности ЙДЗ и действующих программ по их профилактике диктует необходимость проведения соответствующих исследований и мероприятий в отдельных субъектах РФ.ЦЕЛЬ. Провести комплексное исследование по оценке текущей йодной обеспеченности населения Чеченской Республики, проанализировать распространенность тиреоидной патологии и сопоставить с данными официальной статистики, сформулировать выводы о необходимых профилактических мероприятиях.МАТЕРИАЛЫ. В Чеченской Республике всего обследованы 1239 человек, из которых детей допубертатного возраста — 921, взрослого населения — 318. Обследование взрослого населения проводилось на базе медицинских организаций в четырех районах республики (Надтеречный, Шалинский, Веденский, г. Грозный) и включало анкетирование, клинический осмотр эндокринологом с пальпацией щитовидной железы (ЩЖ), УЗИ ЩЖ, исследование качественным методом образцов пищевой соли, используемой в домохозяйствах, на наличие в них йода. Обследование детей проводилось кластерным методом на базе общеобразовательных школ в 9 из 15 районов республики и включало: осмотр эндокринолога и измерение антропометрических параметров (рост, вес), УЗИ ЩЖ для оценки объема, определение концентрации йода в разовых порциях мочи, исследование качественным методом образцов пищевой соли, используемой в питании детей в семьях. Проанализированы заболеваемость и распространенность заболеваний ЩЖ у населения Чеченской Республики с использованием данных официальной государственной статистики — форма №12 «Сведения о числе заболеваний, зарегистрированных у больных, проживающих в районе обслуживания лечебного учреждения» (данные РОССТАТ на 01.01.2021 г.).РЕЗУЛЬТАТЫ. По результатам исследования экскреции йода с мочой у детей (n=921) медианная концентрация йода в моче составляла 71,3 мкг/л (доля образцов мочи с уровнем йода менее 50 мкг/л — 17,7%) и варьировала от 48,9 до 179,2 мкг/л в обследованных районах. По данным УЗИ ЩЖ у 16,4% обследованных детей был выявлен диффузный зоб, частота зоба варьировала от 11,3 до 23,5%. Доля йодированной соли, употребляемой в семьях школьников, во всех районах-исследования составила 4,2% (диапазон значений от 1,3 до 8%), при рекомендованном ВОЗ уровне более 90%.По результатам обследования взрослого населения (n=318) структурные изменения ткани ЩЖ выявлены у 79,9% (n=254) обследованных, при этом доля узловой патологии ЩЖ составила 83% (n=205), диапазон значений по различным районам — 52,5–80%.ЗАКЛЮЧЕНИЕ. На основании полученных данных, в соответствии с критериями ВОЗ, можно констатировать, что в целом по Чеченской Республике степень тяжести ЙДЗ соответствует легкой степени с тенденцией к средней в нескольких районах предгорной местности (Ачхой-Мартановский и Урус-Мартановский). Результатами обследования взрослого населения подтверждена высокая распространенность тиреоидной патологии в регионе, включая сочетание аутоиммунных и узловых форм заболеваний ЩЖ, превышающая данные официальной статистики.Полученные в ходе масштабных исследований данные позволили инициировать разработку необходимых медико-организационных мероприятий в регионе — программу профилактики ЙДЗ.

## Abstract (en)

Iodine deficiency disorders (IDD) are prevalent and highly morbidity, have hidden progression, severe disabling somatic complications, including cognitive disorders, reproductive losses, and oncopathology. This presents a serious challenge to the healthcare system of the Russian Federation, as it affects over 3 million people. The lack of relevant data on the severity of IDD and the current prevention programs at the regional level necessitates the need for appropriate research and measures in individual subjects of the Russian Federation.AIM: To conduct a comprehensive study to assess the current iodine security of the population of the Chechen Republic, to analyze the prevalence of thyroid pathology and compare it with official statistics, to formulate conclusions about the necessary preventive measures.MATERIALS AND METHODS: In the Chechen Republic, a total of 1239 people were examined, of which 921 were schoolchildren of pre-pubertal age (8–10 years) and 318 were adults. The survey of the adult population was carried out at medical organizations in four districts of the republic (Nadterechny, Shalinsky, Vedensky, Grozny) and included a questionnaire survey, a clinical examination by an endocrinologist with palpation of the thyroid gland, thyroid ultrasound, and a study by a qualitative method of samples of table salt used in households for the presence of iodine.Children’s examinations were carried out by the cluster method on the basis of secondary schools in 9 out of 15 districts of the republic and included an examination by an endocrinologist and measurement of anthropometric parameters (height, weight), thyroid ultrasound to evaluate volume, determination of iodine concentration in single portions of urine and qualitative analysis of samples of table salt used in children’s nutrition in families for the presence of iodine.The incidence and prevalence of thyroid disease among the population of the Chechen Republic were analyzed using data from official state statistics — form No. 12 «Information on the number of diseases registered in patients living in the area served by the medical institution» (ROSSTAT data as of 01.01.2021).RESULTS: According to the results of a survey of 921 pre-pubertal children, the median urinary iodine concentration was 71.3 µg/L (frequency of values below 50μg/L — 17,7%) and varies from 48.9 to 179.2 µg/L in the surveyed areas. According to thyroid ultrasound data, diffuse goiter was detected in 16.4% of the examined children, with goiter frequency ranging from 11.3% to 23.5%. The proportion of iodized salt consumed in schoolchildren’s families was 4.2% in all study areas (range of values from 1.3% to 8%), which indicates an extremely low level of using iodized salt by household.According to the results of the examination of the adult population (n=318), structural changes in thyroid tissue were detected in 79.9% (n=254), while the proportion of nodular thyroid pathology being 83% (n=205), with a range of values across different districts of 52.5–80%.CONCLUSION: Based on the obtained data, according to WHO criteria, it can be stated that, overall, the degree of severity of iodine deficiency disorders in the Chechen Republic corresponds to mild severity with a tendency towards moderate severity in several districts of the foothills. The results of the examination of the adult population indicate a high prevalence of thyroid pathology, predominantly nodular, in the Chechen Republic. The data obtained in the course of large-scale research made it possible to initiate the development of necessary medical and organizational measures in the region — a program for the prevention of IDD.

## ВВЕДЕНИЕ

Всемирной организацией здравоохранения (ВОЗ, 2007 г.) дано определение йододефицитных заболеваний (ЙДЗ) — это состояния и нарушения, вызванные йодным дефицитом [1]. ЙДЗ объединяют не только патологию щитовидной железы (ЩЖ), развившуюся вследствие дефицита йода, но и патологические состояния, обусловленные дефицитом тиреоидных гормонов [2][3]. Известно, что наибольшую опасность представляет недостаточное поступление йода в организм на этапе внутриутробного периода и в раннем детском возрасте. Изменения, вызванные ЙДЗ в эти периоды жизни, проявляются необратимыми дефектами в интеллектуальном и физическом развитии детей. Спектр ЙДЗ в различные периоды жизни представлен следующими патологиями (ВОЗ, 2001 г.) [4].

Общими для всех возрастов являются: зоб, гипотиреоз вследствие йодной недостаточности, нарушения когнитивной функции, повышение поглощения радиоактивного йода при ядерных катастрофах.

Исходя их структуры заболеваемости по данным формы №12 Федерального статистического наблюдения, у взрослого населения РФ среди всех случаев эндокринных заболеваний на заболевания ЩЖ приходится до 28% (в 2021 г. всего зарегистрировано заболеваний ЩЖ у взрослых — 3 019 476), уступая по распространенности лишь сахарному диабету, при этом заболеваемость сохраняет свой показатель — доля впервые выявленных случаев составляет 10,4% (число случаев с впервые установленным диагнозом в 2021 г. — 314 388). Обращает на себя внимание высокая распространенность болезней ЩЖ у детей: в 2019 г. она составила 1 439,92 на 100 тыс. д.н.; в 2020 г. — 1 297,59 на 100 тыс. д.н., в 2021 г. доля впервые выявленных случаев заболеваний ЩЖ у детей составляет 26,5%.

За период 2009–2018 гг. в России отмечается статистически значимый рост распространенности ЙДЗ — различных форм зоба, синдрома врожденной йодной недостаточности у всего населения РФ. Медиана распространенности зоба за 10 лет составила 1157,0 случая на 100 000 человек, медиана ежегодного прироста распространенности — 7,5 случая на 100 000 человек. Медиана распространенности врожденной йодной недостаточности за 10 лет составила 3,2 случая на 100 000 человек, медиана ежегодного падения распространенности — 0,1 случая на 100 000 человек [5].

За последние 10 лет в России наблюдается рост распространенности аутоиммунных заболеваний ЩЖ и ассоциированных с ними нарушений функции ЩЖ: распространенность тиреоидита, по данным Росстата на 2021 г., составляет 398,3 случая на 100 000 населения (медиана ежегодного прироста распространенности — 15,9 случая на 100 000 человек); распространенность тиреотоксикоза, по данным Росстата на 2021 г., составляет 128,6 случая на 100 000 населения (медиана ежегодного прироста распространенности — 3,1 случая на 100 000 человек); медиана ежегодного прироста распространенности гипотиреоза — 24,9 случая на 100 000 человек [6]. При этом у лиц старшей возрастной группы отмечен рост заболеваемости узловыми формами зоба с 70,5 до 103,6 случая на 100 тыс. населения, средний ежегодный прирост заболеваемости составил 4%, а также среди лиц старше 60 лет установлена наибольшая заболеваемость тиреотоксикозом — число новых случаев увеличилось с 8,5 до 15,7 на 100 тыс. населения, ежегодный прирост заболеваемости составил 3,4%. Эти тенденции очень опасны в первую очередь тем, что у пожилых людей тиреотоксикоз приводит к обострению ранее существовавших заболеваний сердечно-сосудистой системы, способствует развитию нарушений ритма и повышению риска тpoмбoэмбoлий [7].

Представленные данные наглядно свидетельствуют, что эндемический зоб и другие ЙДЗ представляют собой актуальную медико-социальную проблему, которая может быть решена путем проведения адекватной йодной профилактики. Всеобщее йодирование соли рекомендовано ВОЗ в качестве универсального высокоэффективного и безопасного метода массовой йодной профилактики. Применение йодированной соли во многих случаях способно ликвидировать йодный дефицит в питании населения. Однако в определенные периоды жизни физиологическая потребность в йоде возрастает и организм нуждается в дополнительном поступлении йода. В таких случаях проводится групповая йодная профилактика в группах населения повышенного риска развития ЙДЗ (беременные, кормящие матери, дети до 2 лет) с использованием препаратов йодида калия [8][9].

Многолетний опыт использования йодированной соли в регионах с йодной недостаточностью убедительно показал, что длительная профилактика в итоге снижает частоту тиреотоксикоза в популяции прежде всего за счет устранения факторов, приводящих к формированию многоузловых токсических зобов. Сегодня накапливаются данные и о том, что восполнение дефицита йода влияет на развитие аутоиммунных заболеваний ЩЖ. Таким образом, профилактика дефицита йода — это, по сути, профилактика практически всей патологии щитовидной железы и ее осложнений.

Более 20 лет при поддержке Министерства здравоохранения Российской Федерации ФГБУ «НМИЦ эндокринологии» Минздрава России (ранее — Эндокринологический научный центр) проводит обследование населения страны на предмет выявления распространенности патологий ЩЖ в группах высокого риска их развития с целью планирования и внедрения необходимых профилактических мероприятий с последующим проведением мониторинга их эффективности.

## ЦЕЛЬ

Провести комплексное исследование по оценке текущей йодной обеспеченности населения Чеченской Республики, проанализировать распространенность тиреоидной патологии и сопоставить с данными официальной статистики, сформулировать выводы о необходимых профилактических мероприятиях.

## МАТЕРИАЛЫ И МЕТОДЫ

## Место и время, методы проведения обследования населения, изучаемые популяции.

В Чеченской Республике всего обследованы 1239 человек, из которых — 318 взрослого населения (старше 18 лет) и 921 ребенок допубертатного возраста (7–10 лет).

Обследование взрослого населения проводилось с 28 июня по 1 июля 2022 г. в четырех районах республики: Надтеречный (n=101), Шалинский (n=80), Веденский (n=73), г. Грозный (n=64) и включало сбор анамнеза и анкетирование, осмотр врача-эндокринолога (пальпация ЩЖ, измерение антропометрических показателей (рост, вес)), УЗ-исследование ЩЖ (УЗИ ЩЖ), исследование качественным методом образцов пищевой соли, используемой в домохозяйствах, на наличие в них йода.

В данной когорте пациентов осуществлялся забор биоматериала для лабораторного исследования (определение в сыворотке крови уровней тиреотропного гормона (ТТГ), свободных фракций тироксина и трийодтиронина, антител к тиреопероксидазе) и цитологического анализа пунктата ЩЖ (тонкоигольная аспирационная пункционная биопсия образований ЩЖ выполнялась по показаниям), результаты которых будут представлены в последующих публикациях.

Исследование, направленное на уточнение степени тяжести дефицита йода, проводилось с ноября по декабрь 2022 г. и включало 921 ребенка допубертатного возраста (7–10 лет) с нормальным физическим развитием. Дизайн исследования составлен с учетом рекомендаций ВОЗ [3][4][10][11]: одномоментное популяционное исследование стандартным кластерным методом проводилось на базе общеобразовательных школ в 9 районах региона: Ачхой-Мартановский, Урус-Мартановский, Шатойский, Ножай-Юртовский, Веденский, Наурский, Шелковской, Гудермесский и г. Грозный. В каждом из районов исследования группы были сопоставимы по количеству детей, объем каждой группы был не менее 30 человек. Обследование детей включало сбор анамнеза и измерение антропометрических показателей (рост, вес), осмотр врачом-эндокринологом с пальпацией ЩЖ, оценку объема ЩЖ по данным УЗИ ЩЖ, определение концентрации йода в разовых порциях мочи, исследование качественным методом на наличие йода в образцах пищевой соли, полученных из домохозяйств (образцах соли, используемой в питании детей в семьях).

## Методы

УЗИ ЩЖ выполнялось по стандартной методике в положении лежа с использованием портативного ультразвукового аппарата LOGIQe (China) с мультичастотным линейным датчиком 10–15 МГц. Объем ЩЖ по данным УЗИ ЩЖ рассчитывался с учетом ширины, длины и толщины каждой доли и коэффициента поправки на эллипсоидность Соответствие объема ЩЖ у детей нормативным показателям, разработанным Zimmermann М. и соавт. [12], оценивалось с учетом площади поверхности тела и пола в соответствии с рекомендациями ВОЗ, 1997 (табл. 1).

**Table:** Таблица 1. Нормативные показатели объема щитовидной железы у детей для эпидемиологических исследований (верхний предел нормальных значений — 97 перцентиль)

Площадь поверхности тела, м²	0,8	0,9	1,0	1,1	1,2	1,3	1,4	1,5	1,6	1,7
Мальчики	4,7	5,3	6,0	7,0	8,0	9,3	10,7	12,2	14,0	15,8
Девочки	4,8	5,9	7,1	8,3	9,5	10,7	11,9	13,1	14,3	15,6

Образцы мочи обследуемых переносили в одноразовые пробирки типа Эппендорф и немедленно подвергали заморозке при температуре -20–25°С. Концентрацию йода в моче определяли с помощью церий-арсенитного метода на базе клинико-диагностической лаборатории ФГБУ «НМИЦ эндокринологии» Минздрава России.

Исследование образцов пищевой поваренной соли на наличие йода осуществлялось на месте экспресс-методом качественного анализа. Принцип метода заключается в изменении окраски раствора крахмала при выделении свободного йода из соли после обработки ее тест-раствором, степень изменения окраски оценивается визуально.

Оценка потребления йода населением и степень тяжести ЙДЗ проведена с учетом международных определений и подходов, основываясь на критериях Детского фонда ООН (ЮНИСЕФ), ВОЗ и Глобальной сети по йоду (IGN), представленных в табл. 2.

**Figure:**
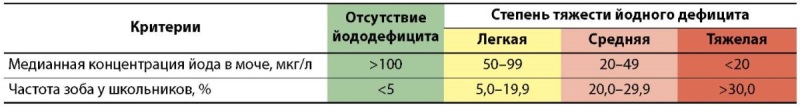
Таблица 2. Эпидемиологические критерии оценки степени тяжести йододефицитных заболеваний

Комментарии. Основным эпидемиологическим показателем статуса йодной обеспеченности населения является медианная концентрация йода в моче (мКЙМ) в репрезентативных группах, наиболее уязвимых к дефициту йода (дети, беременные). Репрезентативной группой принято считать детей младшего школьного возраста (8–10 лет), рекомендовано проводить сбор материала 30-кластерным методом с получением 20–30 образцов в каждом кластере на базе школ, что обеспечивает необходимую рандомизацию [11]. Распространенность зоба по данным УЗИ ЩЖ — косвенный показатель уровня потребления йода, поскольку не отражает текущую ситуацию выраженности йодного дефицита, поэтому не является самостоятельным критерием в отличие от мКЙМ. Определение частоты зоба в популяции имеет значение для оценки степени тяжести йодного дефицита, которое проводится до начала профилактических мероприятий и в последующем используется для оценки и эффективности.

Методом анкетирования была собрана информация от взрослых респондентов, включенных в исследование. Анкета-опросник включала вопросы анамнестического характера, уточняющие факторы наследственности, наличие эндокринной патологии и ряда сопутствующих заболеваний, а также уровень осведомленности о йодном дефиците, рационе питания и употреблении йодированной соли.

Проведен анализ данных официальной государственной отчетности (форма федерального статистического наблюдения №12 «Сведения о числе заболеваний, зарегистрированных у пациентов, проживающих в районе обслуживания медицинской организации»), отражающей абсолютное число случаев заболеваний тиреопатиями всего и впервые выявленных, суммарно у лиц обоего пола всех возрастов, на территории Чеченской Республики по состоянию на 01.01.2021 г. С использованием официальных данных Росстата о численности населения, проживающего на территории региона в соответствующий год, проведен расчет основных эпидемиологических характеристик: распространенности (отношение абсолютного числа случаев заболевания к численности населения, умноженное на 100 000 человек) и заболеваемости (отношение абсолютного числа случаев впервые выявленного заболевания к численности населения, умноженное на 100 000 человек).

## Этическая экспертиза

Протокол исследования одобрен локальным этическим комитетом ФГБУ «НМИЦ эндокринологии» Минздрава России (25 марта 2020 г., №5).

От всех совершеннолетних участников исследования и родителей/опекунов детей получены информированные согласия на проведение обследования и обработку персональных данных.

## Статистический анализ

Обработка и анализ статистических данных проводились в программах MS Excel 2016 (Microsoft, США), Statistica 13 (StatSoft, США). Качественные данные представлены в виде абсолютных значений (n) и/или процентов от общего количества — частот (%). В случае описания количественных показателей, имеющих нормальное распределение, полученные данные объединялись в вариационные ряды, в которых проводился расчет средних арифметических величин (M). Совокупности количественных показателей концентрации йода в моче описывались при помощи значений медианы (Me) и нижнего и верхнего квартилей (Q1–Q3).

По результатам анкетирования взрослого населения сформирована сводка данных о рационе питания и употреблении йодированной соли, уровне информированности о йодном дефиците и способах его профилактики; анализ данных проводился с использованием программы MS Excel.

## РЕЗУЛЬТАТЫ

Чеченская Республика — в числе первых регионов РФ, где в 2022 г. принят в работу проект типовой профилактической программы ЙДЗ, разработанный в ФГБУ «НМИЦ эндокринологии» Минздрава России [13][14], и инициированы исследования для внедрения целевой региональной программы борьбы с ЙДЗ (рис. 1).

**Figure:**
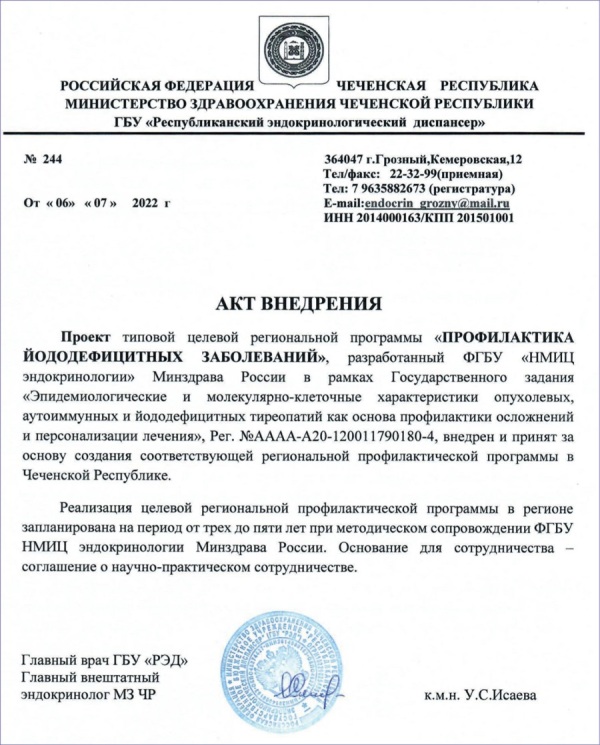
Рисунок 1. Акт внедрения проекта типовой целевой региональной программы «Профилактика йододефицитных заболеваний» в Чеченской Республике.

В рамках Соглашения о сотрудничестве Министерства здравоохранения Чеченской Республики с ФГБУ «НМИЦ эндокринологии» Минздрава России сотрудниками НМИЦ эндокринологии совместно с главным внештатным специалистом эндокринологом региона были организованы и проведены исследования, уточняющие структуру тиреоидной патологии среди взрослого населения и приверженность использования йодированной соли, а также клинико-эпидемиологическое исследование для оценки йодной обеспеченности. Исследований подобного рода в Чеченской Республике ранее не проводилось.

По результатам эпидемиологического исследования экскреции йода с мочой у детей (n=921) медианная концентрация йода в моче (мКЙМ) составила 71,3 мкг/л и варьировала от 48,9 до 179,2 мкг/л в обследованных районах, доля образцов мочи с уровнем йода менее 50 мкг/л — 17,7%. Данные по районам представлены в таблице 3 и на рисунке 2.

**Table:** Таблица 3. Показатели медианы концентрации йода в моче у школьников по районам исследования

Район	Обследовано детей, n	мКЙМ, мкг/л
Ачхой-Мартановский	85	48,89
Ножай-Юртовский	97	67,925
Гудермесский	97	77,6
г. Грозный	183	67,3
Веденский	95	65,59
Наурский	97	122,62
Урус-Мартановский	101	56,17
Шелковской	92	179,185
Шатойский	87	61,91
Итого	921	71,335

**Figure:**
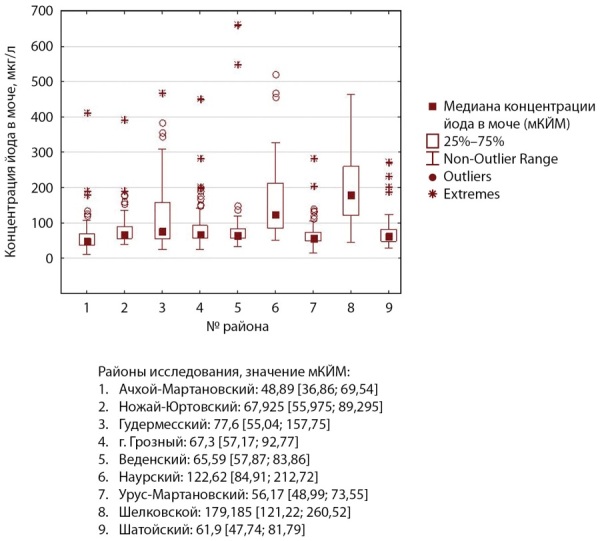
Рисунок 2. Анализ частотного распределения концентрации йода в моче у школьников по районам исследования.

В соответствии с эпидемиологическими критериями, на основании уровня мКЙМ, в целом для Чеченской Республики характерна легкая степень тяжести йодного дефицита. При этом следует отметить, что в ряде районов (Ачхой-Мартановский, Урус-Мартановский) йододефицит соответствовал или был близок к средней степени тяжести.

Во всех районах исследования выявлен крайне низкий уровень употребления йодированной соли в домохозяйствах, установленный экспресс-методом качественного анализа полученных образцов пищевой соли (рис. 3): доля йодированной соли, употребляемой в семьях школьников районов исследования, составила 4,2% (диапазон значений от 1,3 до 8%), при рекомендованном ВОЗ уровне более 90%.

**Figure:**
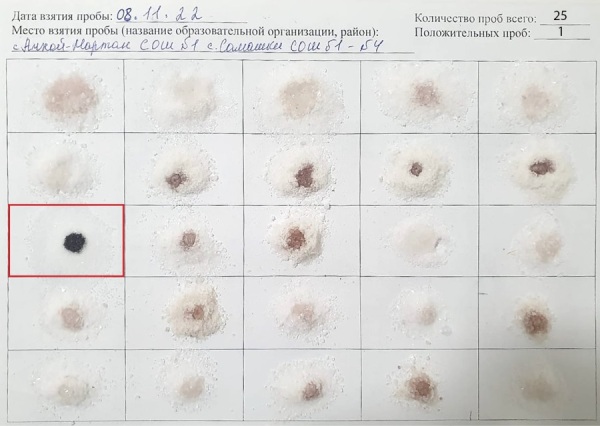
Рисунок 3. Результат исследования экспресс-методом образцов пищевой соли, употребляемой в семьях школьников по районам исследования. Положительная качественная реакция на йод в виде окрашивания темно-синего цвета получена только в 1 одной из 25 проб (ячейка выделена красной рамкой).

По данным УЗИ ЩЖ у 16,4% обследованных детей был выявлен диффузный зоб, частота зоба в исследуемой выборке варьировала от до 11,3 до 23,5%.

Результаты выполненного исследования суммированы в таблице 4, где четко прослеживается и показательна обратная зависимость значений мКЙМ от частоты выявления зобных изменений ЩЖ по данным УЗИ, что подтверждено статистическим анализом данных — получена сильная корреляционная связь (R=0,9) этих показателей.

**Figure:**
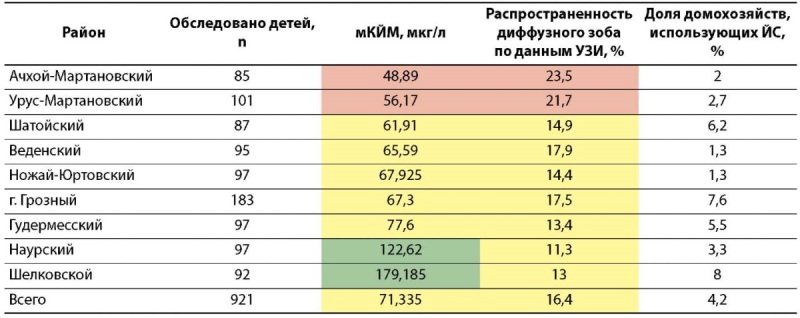
Таблица 4. Показатели медианы концентрации йода в моче (мКЙМ), распространенности диффузного зоба у детей и доли домохозяйств, использующих йодированную соль (ЙС) в районах Чеченской Республики

Среди всех районов исследования, указанных в таблице 4, выделяются первые два — Ачхой-Мартановский и Урус-Мартановский, в которых были установлены самые низкие показатели мКЙМ (48,9 и 56,17 мкг/л соответственно) и наибольшая частота распространенности зоба у детей (23,5 и 21,7% соответственно). Согласно критериям ВОЗ, эпидемиологическая ситуация в указанных районах Чеченской Республики расценивается как йодный дефицит средней степени тяжести.

В 2 из 9 районов исследования зафиксирована мКЙМ >100 мкг/л — соответствует адекватной йодной обеспеченности, при этом уровень распространенности зоба у детей значимо превышает пороговое значение спорадической его встречаемости (критерий ВОЗ <5%): Наурский — 11,3% и Шелковской — 13%, что в совокупности характеризуется практически полным отсутствием использования йодированной соли в домохозяйствах и не позволяет расценивать эпидемиологическую ситуацию как благополучную.

Анализ результатов в разрезе эколого-географических зон региона наглядно представляет районы с наибольшей степенью выраженности йодного дефицита в предгорной и горной местностях по сравнению с равнинными районами (табл. 5).

**Figure:**
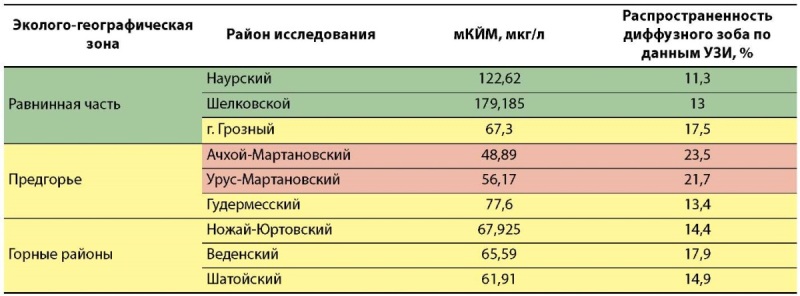
Таблица 5. Показатели медианы концентрации йода в моче (мКЙМ) и распространенности диффузного зоба у школьников в различных эколого-географических зонах Чеченской Республики

Таким образом, можно констатировать, что в целом по Чеченской Республике степень тяжести ЙДЗ соответствует легкой степени тяжести на основании мКЙМ, равной 71,335 мкг/л, и распространенности диффузного зоба у детей — 16,4%, с выделением нескольких районов предгорной местности (Ачхой-Мартановский и Урус-Мартановский), где выраженность йодного дефицита соответствует средней степени тяжести.

С целью оценки распространенности и структуры патологии ЩЖ в Чеченской Республике также проведено обследование 318 условно здоровых лиц (18 лет и старше). Характеристика группы взрослого населения: средний возраст — 47,5 года (от 18 до 76 лет, медианный интервал возраста — 48 лет), доля женщин составила 89,6%.

По результатам анкетирования (n=294) доля взрослого населения республики, знающего про дефицит йода и его последствия для здоровья, составила 57%, при этом утвердительные ответы об исключительно либо преимущественно регулярном использовании йодированной соли в питании были получены от 26,7% респондентов. Методом качественного анализа было подтверждено наличие йода в пищевой соли только в 11,7% образцов (n=294), что отражает истинную долю домохозяйств с йодированной солью в питании.

Результатами УЗ-скрининга подтверждена высокая распространенность тиреоидной патологии у взрослого населения: структурные изменения ткани ЩЖ выявлены у 79,9% (n=254) обследованных, при этом доля узловой патологии ЩЖ составила 83% (n=205) (диапазон значений по различным районам 52,5–80%). Сочетание эхографических признаков узловой патологии ЩЖ и аутоиммунного тиреоидита встречается в 12,6% (n=32) случаев общей распространенности структурных изменений ЩЖ. Полученные данные обследования взрослого населения региона по распространенности тиреоидной патологии превышают официальную статистику и отражены в табл. 6.

**Table:** Таблица 6. Данные обследования взрослого населения Чеченской Республики в сопоставлении с данными форм официальной статистической информации

Обследовано, человек	Структурные изменения ЩЖ по данным УЗИ	из них, узловой зоб	из них, сочетание узлового зоба и признаков аутоиммунного тиреоидита	Официальная статистика (Росстат, 2021)
Заболевания ЩЖ на 100 тыс. нас.	из них, нетоксический зоб (все формы)	из них, тиреоидит
318	79,9%	83%	12,6%	28% (25 559)	73,6% (18 827)	8,8% (2264)

По результатам анализа форм официальной статистической информации, Чеченскую Республику отличает более высокая распространенность тиреоидной патологии в структуре болезней эндокринной системы как среди взрослого населения (43,2%), так и у детей (41%), чем в целом по стране (28% у взрослых, 25% у детей), охватывая 30,4 тыс. населения республики (табл. 7).

**Table:** Таблица 7. Структура болезней эндокринной системы среди населения Чеченской Республики по данным Росстата на 01.01.2021 г.

01.01.2021 г.	Зарегистрировано заболеваний,взрослые 18 лет и более	Зарегистрировано заболеваний,дети 0–17 лет
Код по МКБ-10	Наименование классов и отдельных болезней	всего	из нихс впервые в жизни установленным диагнозом	всего	из нихс впервые в жизни установленным диагнозом
Е00–Е89	Болезни эндокринной системы, расстройства питания и нарушения обмена веществ	59 050	8957	11 914	1217
Е00–Е07	из них: болезни щитовидной железы	25 559 (43,2%)	3585 (40%)	4886 (41%)	716 (%)
Е00	из них: синдром врожденной йодной недостаточности	14	-	13	1
Е01.0–2	эндемический зоб, связанный с йодной недостаточностью	11 937	1673	2991	662
Е02, Е03	субклинический гипотиреоз вследствие йодной недостаточности и другие формы гипотиреоза	3400	522	36	13
Е04	другие формы нетоксического зоба	6890	951	26	6
Е05	тиреотоксикоз (гипертиреоз)	1054	123	11	1
Е06	тиреоидит	2264	301	13	0

Среди заболеваний ЩЖ у населения Чеченской Республики наибольшая доля приходится на эндемический и другие формы нетоксического зоба, составляя 73,6% у взрослых (18 827 случаев на 01.01.2021, Росстат) и 61,8% у детей (3023 случая на 01.01.2021, Росстат), что также превышает общероссийские показатели у взрослых и детей — 48,3 и 57,7% соответственно. Общая распространенность патологии ЩЖ в Чеченской Республике составляет 2699 случаев на 100 тыс. взрослого населения и 886,7 случая на 100 тыс. детского населения, распространенность эндемического и других форм нетоксического зоба — 1988 случаев на 100 тыс. взрослого населения и 547,5 случая на 100 тыс. детского населения.

По состоянию на 01.01.2021 г. в Чеченской Республике всего зарегистрировано 16 339 заболеваний врожденным гипотиреозом (распространенность составляет 1090,7 случая на 100 тыс. населения), в целом по РФ зарегистрировано 1 229 676 случаев (распространенность — 841,2 случая на 100 тыс. населения). Уровень ТТГ у новорожденных является индикатором выявления неонатального гипотиреоза и чувствительным маркером йодного дефицита при использовании определенных пороговых значений уровня ТТГ. В соответствии с рекомендациями ВОЗ в рамках эпидемиологических исследований на достаточную обеспеченность населения йодом указывает доля новорожденных менее 3% с концентрацией неонатального ТТГ выше 5 мЕд/л, однако эти данные следует получать отдельным запросом в медико-генетических лабораториях и анализировать в сопоставлении с данными по мКЙМ.

Сохраняющаяся стойкая ситуация с выявлением случаев врожденного гипотиреоза объясняется не только возрастающей долей охвата новорожденных скринингом (табл. 8), а в том числе недостаточными и неадекватными мерами профилактики йодного дефицита у беременных.

**Table:** Таблица 8. Данные первичного теста неонатального скрининга на врожденный гипотиреоз (ВГ) в Чеченской Республике по годам (предоставлены медико-генетической лабораторией региона) * — диагноз врожденного гипотиреоза устанавливался при выявлении уровня ТТГ более 50 мЕд/л по результатам первичного теста.

Год	2017	2018	2019	2020	2021	2022
Обследовано на ВГ	22105	25450	26010	27000	8450	26350
Охват скринингом, %	73,95	84,98	87,24	89,66	27,84	90,86
Выявлен ВГ*	1	4	4	4	1	3

## ОБСУЖДЕНИЕ

Следует отметить масштабность впервые проведенного в регионе клинико-эпидемиологического исследования по оценке йодной обеспеченности, спланированного на основе принципов кластерного метода, с включением 9 из 15 районов региона и учетом представленности всех эколого-географических зон территории, что позволяет составить достаточно полную картину по республике.

Оценка выраженности йодного дефицита при проведении эпидемиологического исследования осуществлялась на основании ключевых параметров: экскреция йода с мочой, частота распространенности зоба и доля населения, потребляющего йодированную соль. Анализируя полученные данные (табл. 7), первостепенного внимания среди всех районов исследования требуют Ачхой-Мартановский и Урус-Мартановский районы, в которых установлен йодный дефицит средней степени тяжести (мКЙМ 48,9 и 56,17 мкг/л соответственно и частота распространенности зоба 23,5 и 21,7% соответственно).

Отдельный интерес представляют другие 2 из 9 районов исследования (табл. 7) с адекватной йодной обеспеченностью по результатам определения медианы концентрации йода в моче (мКЙМ>100 мкг/л), при этом уровень распространенности зоба у детей более чем в 2 раза превышает спорадическую встречаемость: Наурский — 11,3% и Шелковской — 13% (критерий ВОЗ<5%). Этот результат свидетельствует об относительно недавнем улучшении йодной обеспеченности, поскольку увеличение щитовидной железы формируется в условиях продолжительного воздействия йодного дефицита и меняется спустя достаточно длительный срок после нормализации потребления йода. Наиболее вероятно, в этих равнинных районах с возможно менее выраженным природным йододефицитом, экскреция йода с мочой у детей улучшилась благодаря соблюдению с 2020 г. СанПиН 2.4.5.2409–08, предусматривающего обязательное использование йодированной соли в школьном питании, но это не позволяет экстраполировать полученные данные на всю популяцию указанных районов и считать адекватной йодную обеспеченность в других группах риска — среди беременных и кормящих.

Значительное снижение числа стран с дефицитом йода за последние 25 лет было обусловлено массовым внедрением программ всеобщего йодирования соли во всем мире [5][15]. В настоящее время йодирование всей соли для использования в пищевых целях в домохозяйствах и в пищевой промышленности расценивают как наиболее эффективную и устойчивую стратегию массовой профилактики и устранения нарушений, вызванных дефицитом йода, у населения [5]. Обеспечение всеобщей доступности адекватно йодированной соли должно, таким образом, оставаться важной целью программ профилактики и достигается при охвате более 90% домохозяйств йодированной солью [4]. По результатам анкетирования взрослого населения Чеченской Республики, проведенного в рамках данного исследования, утвердительные ответы о регулярном использовании йодированной соли в питании были получены от 26,7% респондентов, что в более чем 2 раза отличается от истинной доли домохозяйств с йодированной солью в питании (11,7%). Таким образом, по результатам исследования отмечаются низкая информированность населения о проблеме йододефицита (осведомлены только 57%) и еще более низкая приверженность респондентов к проведению массовой йодной профилактики, что в целом согласуется с данными других опросов населения РФ [16]. Этот анализ также демонстрирует, что в рамках эпидемиологических исследований и для мониторинга эффективности программ профилактики ЙДЗ необходимо использовать методики качественной или количественной оценки содержания йода в пищевой соли, а не данные опросов населения.

Сопоставление мКЙМ и доли потребления йодированной соли с пищей (в том числе в составе продуктов питания промышленного производства) у определенных групп населения лежит в основе принятия решений о необходимости внесения изменений в программу йодной профилактики и должно быть отдельным направлением для исследований и мониторинга предпринимаемых профилактических мероприятий в регионе.

В условиях отсутствия Федерального закона о профилактике заболеваний, связанных с дефицитом йода, исключительно актуальным шагом на пути решения задачи по борьбе с ЙДЗ должно стать формирование единого в масштабах страны профилактического процесса, базирующегося на соответствующей нормативно-правовой базе в каждом субъекте РФ [12]. С целью способствования эффективной реализации «Стратегии формирования здорового образа жизни населения, профилактики и контроля неинфекционных заболеваний на период до 2025 года», утвержденной Приказом Минздрава России от 15.01.2020 №8, в 2022 г. экспертами ФГБУ «НМИЦ эндокринологии» Минздрава России разработан проект типовой региональной целевой программы профилактики ЙДЗ [13], который после проведения необходимых исследований может быть адаптирован и внедрен в каждом субъекте РФ.

## Ограничения исследования

Некоторым ограничением исследования явилось смещение выборки в сторону преобладания женщин (более 80%), поскольку исследование проводилось исключительно на добровольной основе и учитывались только результаты собственных лабораторно-инструментальных исследований. Среди недостатков исследования следует отметить невозможность сопоставления частоты встречаемости коморбидных заболеваний ЩЖ (узловых форм зоба и аутоиммунных поражений), выявленных по результатам обследования в сравнении с данными официальной статистики, что обусловлено исходными данными статистической отчетности.

## Направления дальнейших исследований

Адекватность потребления йода следует оценивать среди разных сегментов населения, наиболее уязвимых к дефициту йода. Дефицит йода оказывает неблагоприятное воздействие на рост и развитие организма еще во внутриутробном периоде, что проявляется нарушением формирования и развития нервной системы плода, прежде всего — головного мозга, с ограничением когнитивных и функциональных параметров развития у детей, приводит к снижению уровня интеллекта (показатель IQ) вплоть до выраженных форм умственной отсталости, повышенной частоте врожденных пороков развития и невынашивания беременности. В связи с этим, при согласовании с Министерством здравоохранения Чеченской Республики, в продолжение работы планируется проведение исследований с включением беременных и кормящих женщин.

Дополнительно, для дальнейшей оценки степени выраженности ЙДЗ и мониторинга предпринимаемых профилактических мероприятий в регионе, рекомендуется системное проведение анализа результатов неонатального скрининга и определение частоты выявления концентрации ТТГ >5 мкЕД/мл.

За последнее десятилетие значимо возросли доля употребления промышленно переработанных пищевых продуктов (ПППП), что обусловлено процессом урбанизации, равно как и использование йодированной соли в ПППП. Такие продукты могут быть важным источником йода в питании, когда использование йодированной соли в домашних хозяйствах невелико. Поэтому оценка потребления йода населением с ПППП, выделение основных пищевых источников поступления йода с рационом питания, особенно в группах риска развития ЙДЗ, также является важным направлением для дальнейших исследований и обеспечит дополнительными сведениями для повышения эффективности проводимых профилактических мероприятий.

## ЗАКЛЮЧЕНИЕ

Чеченская Республика относится к регионам с природным йодным дефицитом. На основании результатов проведенного впервые на территории Чеченской Республики масштабного эпидемиологического исследования, в соответствии с критериями ВОЗ, установлено, что в целом для Чеченской Республики степень тяжести ЙДЗ соответствует легкой степени тяжести с тенденцией к усугублению ситуации в предгорных и горных районах, в ряде которых степень тяжести йодного дефицита соответствует средней.

Значимость полученных результатов для системы здравоохранения состоит в комплексной оценке состояния йодной обеспеченности и зобной эндемии, необходимости принятия целевых региональных программ профилактики ЙДЗ на территории Чеченской Республики.

Результаты исследования демонстрируют высокую медико-социальную актуальность проблемы ЙДЗ в Чеченской Республике. Данные по распространенности ЙДЗ среди взрослого населения и оценка йодной обеспеченности, детализированная порайонно и в разрезе географических зон, послужат необходимым базисом при разработке региональной целевой программы профилактики ЙДЗ и позволят региональным органам здравоохранения проводить последующий мониторинг эффективности программы.

## ДОПОЛНИТЕЛЬНАЯ ИНФОРМАЦИЯ

Источник финансирования. Исследование с целью проведения обследования взрослого населения Чеченской Республики на предмет распространенности патологии ЩЖ выполнено по гранту РНФ «Научное обоснование, разработка и внедрение новых технологий диагностики коморбидных йододефицитных и аутоиммунных заболеваний щитовидной железы, в том числе с использованием возможностей искусственного интеллекта» (проект №22-15-00135). Обследование детской когорты выполнено за счет ресурсов и средств региона, разработка концепции дизайна эпидемиологического исследования и формирование рекомендаций для разработки региональной программы «Профилактика йододефицитных заболеваний» проведены в рамках целевого финансирования на выполнение научно-исследовательской работы (НИР) «Эпидемиологические и молекулярно-клеточные характеристики опухолевых, аутоиммунных и йододефицитных тиреопатий как основа профилактики и персонализации лечения» (рег. №АААА-А20-120011790180-4).

Конфликт интересов. Авторы декларируют отсутствие явных и потенциальных конфликтов интересов, связанных с публикацией настоящей статьи.

Участие авторов. Все авторы одобрили финальную версию статьи перед публикацией, выразили согласие нести ответственность за все аспекты работы, подразумевающую надлежащее изучение и решение вопросов, связанных с точностью или добросовестностью любой части работы.

Благодарности. Авторы выражают признательность Министерству здравоохранения Чеченской Республики в лице министра здравоохранения Лорсанова Сулеймана Майрбековича и заместителя министра Фадеева Павла Александровича за всестороннюю поддержку в подготовке и проведении исследования, отдельно — главному внештатному специалисту эндокринологу Минздрава Чеченской Республики Исаевой Умулкулсум Султановне за непосредственное участие во всех организационных и клинических этапах работы, а также благодарят руководителей и сотрудников медицинских организаций в районах проведения исследования.
